# A Case of Asymptomatic Perforated Gangrenous Cholecystitis in a Diabetic Patient: A Critical Condition

**DOI:** 10.7759/cureus.48014

**Published:** 2023-10-30

**Authors:** Elias E Lahham, Abdalrazeq A Ghweir, Qusai A Alsalah, Mohammad I Alsahouri, Mohammad AlQadi

**Affiliations:** 1 Department of Radiation Oncology, Augusta Victoria Hospital, Jerusalem, PSE; 2 Faculty of Medicine, Palestine Polytechnic University, Hebron, PSE; 3 Department of General Surgery, Beit-Jala Governmental Hospital, Bethlehem, PSE

**Keywords:** case report, gangrenous cholecystitis, subtotal cholecystectomy, cholelithiasis, cholecystitis

## Abstract

Gangrenous cholecystitis (GC) is a severe form of acute cholecystitis (AC) with ischemia and necrosis of the gallbladder (GB) wall. Patients with GC are sicker than the usual AC patients, and their surgical treatment is more complex and linked with a higher risk of morbidity and mortality. Typically, the first imaging modality used to assess patients with clinically suspected AC is ultrasound. However, if the ultrasound results were inconclusive, a CT scan might help evaluate these individuals. Our study presents a 62-year-old male who presented with mild right upper quadrant discomfort. However, an abdominal computed tomography CT scan showed a pericholecystic fluid collection with a sign of GB perforation that was managed with subtotal cholecystectomy. Five days after the operation, the patient was discharged to home in excellent condition.

## Introduction

Acute cholecystitis (AC) is the most frequent complication of cholelithiasis and is a serious and life-threatening condition [1]. The presence of gallstones associated with RUQ pain and fever highly supports the diagnosis of AC [2,3]. However, roughly 1-2% of patients may remain asymptomatic [1]. AC may develop into GC, first described in 1894 and defined as a severe form of AC with ischemia and necrosis of the GB wall [4]. Patients with GC are sicker than the usual AC patients, and their surgical treatment is more difficult and linked with a higher mortality rate [5]. GC risk factors include coronary heart disease, diabetes mellitus, older age, males, and elevated white blood cells [3,5,6]. The first-line imaging modality is ultrasound. However, if it is inconclusive, a CT scan can help [3]. There have been reports of postperforation reduction of abdominal pain due to potential nerve denervation, particularly in diabetic individuals with neuropathy [3,7]. Our study presents a 62-year-old diabetic male patient who presented with mild RUQ abdominal discomfort that resolved over time; due to our caution, we ordered an abdominal CT scan which is consistent with perforated GC with localized fluid collection in the subcapsular space. Therefore, the patient was managed successfully with subtotal cholecystectomy. This report aims to shed light on this rare entity and alerts clinicians to have a low threshold to conduct a CT scan of the abdomen, especially in diabetic patients with vague or minimal symptoms.

## Case presentation

**Figure 1:**
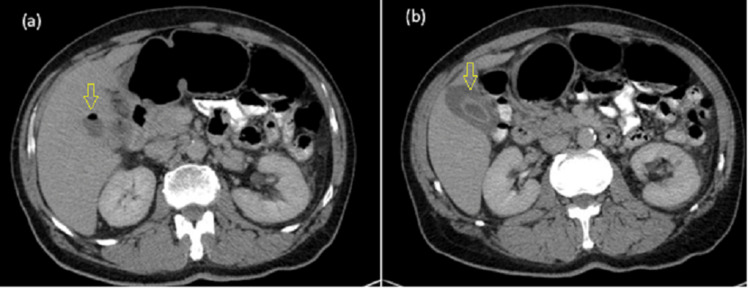
Preoperative abdominal CT with contrast shows (a) air-filled gallbladder and (b) the gallbladder revealed wall thickening and focal mural disruption, with a pericholecystic fluid collection

**Figure 2:**
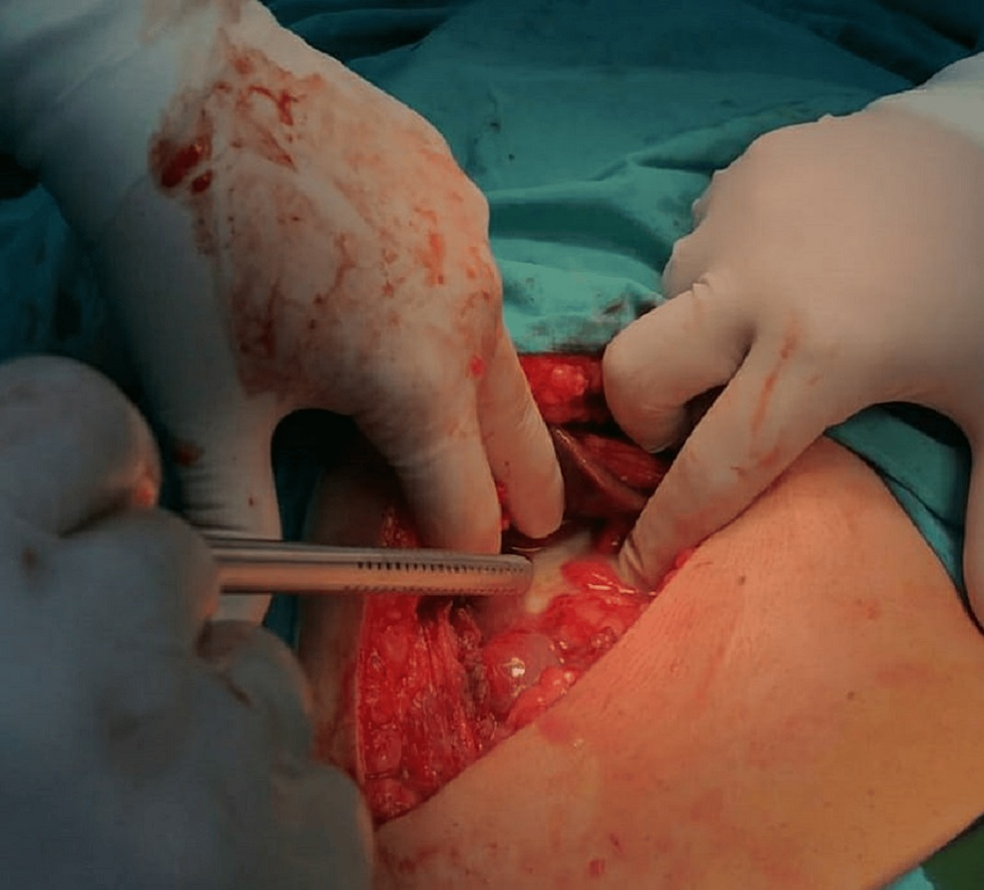
Intraoperative image A Kocher incision was done. Intraoperatively, there were severe abdominal adhesions, a ruptured gallbladder, subhepatic pus, and the absence of identifiable structures in Calot's triangle (frozen Calots) prompted a choice to proceed with a subtotal cholecystectomy.

A 62-year-old male patient with a history of hypertension and diabetes mellitus type 2 presented to our department with RUQ abdominal discomfort for three days, associated with nausea, with no history of vomiting, loss of appetite, abdominal distention, or change in bowel habits. On admission, the patient had stable vital signs and was afebrile. Physical examination revealed mild RUQ tenderness. However, Murphy’s sign was negative. The abdomen was soft and lax. No scars, hernias, or masses were found. Laboratory workup showed a high white blood cell count (15 cells/l) and C-C-reactive protein (CRP) (230 g/dl). The liver function test was normal. A chest X-ray was normal, with no subdiaphragmatic air. An abdomen ultrasound (U/S) revealed a pericholecystic fluid, nondistended thick gallbladder (GB) (wall thickness 4mm), and stones impacted in the GB neck with subhepatic fluid collection, suggesting the diagnosis of AC with micro perforation. Additionally, a CT scan of the abdomen with contrast was performed (Figure 1), which showed an image compatible with acute perforated GC, with subcapsular fluid collection. With these findings, the decision to perform an urgent surgical intervention was made. IV fluids and antibiotics were started. Under general anesthesia, a Kocher incision was done. Intraoperatively, severe abdominal adhesion, GB perforation, subhepatic pus, and Friable tissue were found (Figure 2).

**Figure 3:**
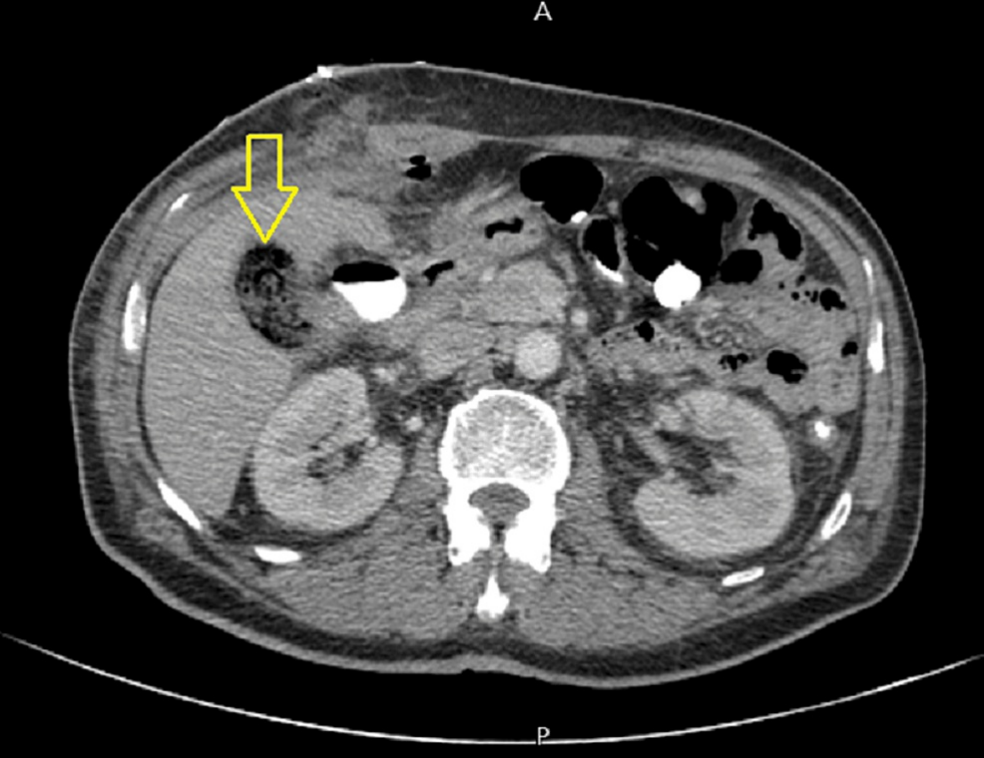
Postoperative abdominal CT with contrast shows mixed gas–fluid attenuation in gallbladder fossa consistent with surgical (normal) findings

Obliteration of Calot's triangle anatomy was observed. The cystic duct, cystic artery, and common hepatic duct were not identifiable (frozen Calots), so the decision was to proceed with subtotal cholecystectomy. Two surgical drains were inserted, one in the pelvis and the other in the subhepatic space; both were removed on day 4 after the surgery. CT control on day 3 after the surgery showed no leakage or fluid collections (Figure 3).

The postoperative course was uneventful; the patient was started on a soft diet on day 2 and discharged on day 6 after the operation. Histopathologic examination confirmed the diagnosis of acute on top of chronic cholecystitis with necrotic tissue. 

## Discussion

AC in patients might develop into GC in around 2-29.6% of cases [1]. It results from the GB's noticeable distension, which raises the GB wall's tension. Ischemic necrosis of the wall is caused by concomitant inflammation, which may or may not be accompanied by cystic artery thrombosis [5]. Due to neuropathy, which prevents them from feeling pain, patients with long-term diabetes may be asymptomatic. In other cases, diabetic individuals who initially underwent a benign abdominal exam were later found to have GC, either on imaging or during surgery [8]. Nearly 22% of the mortality and 25% of complication rates have been linked to GC [1]. Perforation has been found to happen in as many as 10% of instances of AC [5], which may be complicated by a subsequent abscess or peritonitis [5,9]. Bilious fluid may accumulate around the liver and grow to the area between the Glisson's capsule and the liver itself, known as the subcapsular space [3]. Therefore, unlike AC, it is critical to correctly detect and surgically treat GC before it develops complications or perforations to reduce its high morbidity and mortality rates [10]. Typically, the first imaging modality used to assess patients with clinically suspected AC is ultrasound, which showed the presence of striated thickening of the GB wall and pericholecystic fluid collections [11]. However, if the sonography results are unclear, a CT scan might help evaluate these individuals. A pericholecystic abscess, intraluminal membranes, an uneven wall, and gas in the wall or lumen are the CT findings most specific for acute GC [12]. The mainstay of the treatment is surgical intervention. However, due to chronic inflammation and adhesions, it can be challenging to visualize Calot's triangle [13]. Fortunately, subtotal cholecystectomy was used to treat our patient effectively. Madding's introduction of the open subtotal cholecystectomy in 1955 provided a safe replacement for total cholecystectomy by preventing incorrect cystic duct identification [14]. In our case, the patient initially displayed some characteristics of AC, including RUQ abdominal discomfort, which eventually went away. We proceeded with care and underwent abdominal CT imaging when we discovered the patient had a perforated GC.

## Conclusions

GB perforation is a life-threatening condition that can be masked in a patient with diabetic neuropathy, so in these patients, the surgeon should lower the threshold for CT imaging and raise the suspicion of severe intra-abdominal disease even in the setting of mild abdominal pain, as in our case. Early diagnosis and management are essential in cases of perforated GB to decrease morbidity and mortality. Subtotal cholecystectomy could be a surgical option in cases where it is challenging to identify Calot's triangle and the presence of severe abdominal adhesion.

## References

[1] Medina VJ, Martial AM, Chatterjee T (2023). Asymptomatic gangrenous acute cholecystitis: a life-threatening condition. Cureus.

[2] Aleman Espino E, Kazaleh M, Zaglul J, Frontela O (2023). Acute cholecystitis presenting with atypical radiologic or laboratory findings: a case report. Cureus.

[3] Faraji M, Sharp R, Gutierrez E, Malikayil K, Sangi A (2020). Perforated gangrenous gallbladder in an asymptomatic patient. Cureus.

[4] Hotchkiss LW (1894). Gangrenous cholecystitis. Ann Surg.

[5] Chaudhry S, Hussain R, Rajasundaram R, Corless D (2011). Gangrenous cholecystitis in an asymptomatic patient found during an elective laparoscopic cholecystectomy: a case report. J Med Case Rep.

[6] Hunt DR, Chu FC (2000). Gangrenous cholecystitis in the laparoscopic era. Aust N Z J Surg.

[7] Mehrzad M, Jehle CC, Roussel LO, Mehrzad R (2018). Gangrenous cholecystitis: a silent but potential fatal disease in patients with diabetic neuropathy. A case report. World J Clin Cases.

[8] Merriam LT, Kanaan SA, Dawes LG, Angelos P, Prystowsky JB, Rege R V, Joehl RJ (1999). Gangrenous cholecystitis: analysis of risk factors and experience with laparoscopic cholecystectomy. Surgery.

[9] Grant RL, Tie ML (2002). False negative biliary scintigraphy in gangrenous cholecystitis. Australas Radiol.

[10] Dhir T, Schiowitz R (2015). Old man gallbladder syndrome: gangrenous cholecystitis in the unsuspected patient population. Int J Surg Case Rep.

[11] Katsumata R, Manabe N, Urano T (2022). Asymptomatic gangrenous cholecystitis diagnosed using contrast-enhanced ultrasonography in a patient with pancreatic cancer. Radiol Case Rep.

[12] Bennett GL, Rusinek H, Lisi V, Israel GM, Krinsky GA, Slywotzky CM, Megibow A (2002). CT findings in acute gangrenous cholecystitis. AJR Am J Roentgenol.

[13] Ashfaq A, Ahmadieh K, Shah AA, Chapital AB, Harold KL, Johnson DJ (2016). The difficult gall bladder: outcomes following laparoscopic cholecystectomy and the need for open conversion. Am J Surg.

[14] Segal MS, Huynh RH, Wright GO (2017). Case report: modified laparoscopic subtotal cholecystectomy: an alternative approach to the "difficult gallbladder". Am J Case Rep.

